# Blood‐based biomarkers enrich for amyloid positive participants, reducing patient burden in PROGRESS‐AD, a phase 2 clinical trial for early Alzheimer's disease

**DOI:** 10.1002/alz70856_103351

**Published:** 2025-12-25

**Authors:** Rianne N Esquivel, Shriya Mathur, Christine Parker, Brady Burgess, Gopinath Ganji, Michael Boss, Jeffrey Lin, Sabine Lenton, Lovingly Park, Christine Bailey, Robert Y. K. Lai

**Affiliations:** ^1^ GSK, Collegeville, PA, USA; ^2^ GSK, Stevenage, Hertfordshire, United Kingdom; ^3^ Alector, South San Francisco, CA, USA

## Abstract

**Background:**

Enrollment in clinical trials for early Alzheimer's disease is typically slow due to low rates of amyloid positivity but essential confirmatory testing using CSF biomarkers or amyloid PET imaging increases patient burden and operational challenges. By prescreening participants with blood biomarkers to enrich for amyloid positivity, enrollment can be accelerated, making trials more accessible and reducing overall costs.

**Methods:**

We incorporated PrecivityAD (C2N Diagnostics) and Elecsys pTau181 (Roche Diagnostics) assays into the ongoing screening for PROGRESS‐AD (EU CT: 2023‐505083‐11‐00; NCT06079190; GSK Study ID 219867), a phase 2 double‐blind study enrolling approximately 282 patients with early Alzheimer's disease to receive anti‐sortilin mAb, GSK4527226/AL101, or placebo. Plasma samples from participants were tested and were considered amyloid positive for PrecivityAD APS score ≥ 36 (includes both intermediate and high range) or pTau181 ≥1 pg/ml. These results were compared to amyloid PET and CSF (Lumipulse β‐amyloid (1‐42/1‐40) ratio). Historical results were acceptable utilizing FDA approved tracers or CSF ratios with tests that meet regional clinical and regulatory requirements. Confirmatory PET or CSF results were available for PrecivityAD (*n* = 172) and pTau181 (*n* = 190) for ROC analysis with and without Beggs and Greene correction. Human biological samples were sourced ethically, and their research use was in accord with terms of the informed consents under an IRB/REC approved protocol.

**Results:**

For pTau181, the positive percent agreement (PPA) and negative percent agreement (NPA) with comparators were 85.0% [78.4 – 89.8] and 59.5% [42. – 75.] respectively. PrecivityAD had a PPA and NPA of 64.6% [57.1‐71.5] and 42.9% [15.8 – 74.9] respectively. The positive predictive value (PPV) for participants using PrecivityAD was 96.4% compared to the PPV of 90.0% from participants using pTau181. Overall, this reduced the screen failure rate to confirm amyloid positivity to 9.9%.

**Conclusions:**

Utilizing blood‐based biomarkers significantly decreased the number of participants required to have unnecessary CSF or PET testing. Limitations of this study are incomplete confirmatory results for amyloid negative individuals, regional PET/CSF confirmatory test bias, and the delay between historical result and blood biomarker testing. Including blood‐based biomarkers in screening can reduce participant burden, enhance access and enrollment and improve clinical trial design.

